# An unusual cause of haemoptysis in a young male

**DOI:** 10.1186/1477-7800-3-6

**Published:** 2006-03-07

**Authors:** N Barbetakis, A Efstathiou, T Xenikakis, H Konstantinidis, I Fessatidis

**Affiliations:** 1 Cardiothoracic Surgery Department, Geniki Kliniki – Euromedica, Thessaloniki, Greece

## Abstract

Inflammatory myofibroblastic tumours are reported to occur in a variety of sites, including the head and neck, abdominal organs, central nervous system and urinary tract. They only rarely occur in the lung.

We report a case of a 25-year-old male admitted with haemoptysis. His chest radiograph showed a peripheral right lung opacity and computed tomography revealed a right lower lobe soft tissue density mass. Bronchoscopy and fine needle aspiration were unhelpful. a diagnosis of pulmonary carcinoma was made, and the patient underwent a right lower lobectomy. On pathology, the tumor was found to be an inflammatory pseudotumor. These lesion are extremely rare, constituting less than 1% of pulmonary malignancies, but are known to occur in young patients. We believe clinicians need to retain an index of suspicion for the presence of this disease in young patients, which can masquerade as more common malignancies.

## Introduction

Inflammatory myofibroblastic tumor (IMT) is a rare disease that usually occurs in the lung. It is also known as plasma cell granuloma, inflammatory pseudotumor, xanthogranuloma and fibrous histiocytoma [1]. The notion of IMT being a reactive lesion or a neoplasm is controversial [2]. Because of its rarity, its biologic nature, natural history and response to treatment have yet to be completely defined. A case of a 25-year-old male who was admitted with hemoptysis, right lung mass and underwent a right lower lobectomy is presented. Biopsy of the resected specimen confirmed the diagnosis of an IMT.

## Case presentation

A 25 year-old-male presented with a history of cough with bloody mucoid sputum for the previous two months. He also had anorexia and a history of intermittent upper respiratory tract infections for two years. Clinical examination revealed diminished breath sounds at the right lung base. Laboratory investigation reported normochromic – normocytic anaemia, with low iron serum level and elevated erythrocyte sedimentation rate (76 mm/first hour).

**Figure 1:**
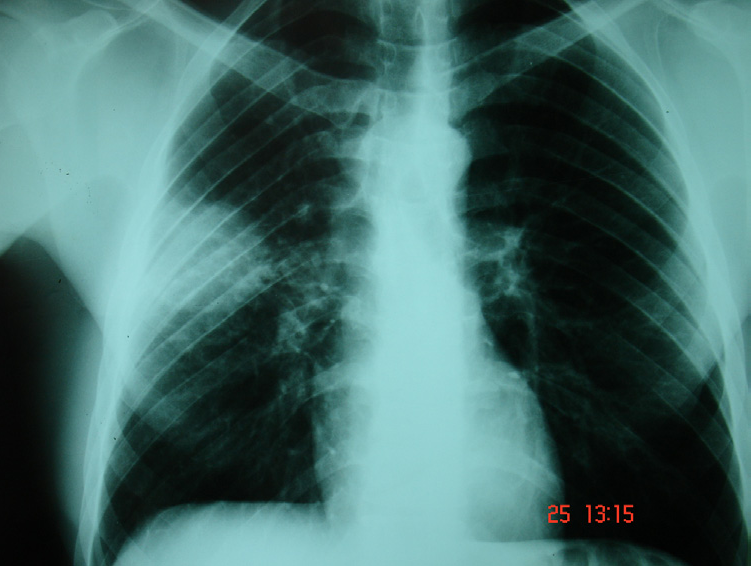
Chest x-ray revealed an abnormal shadow on the right lung.

**Figure 2:**
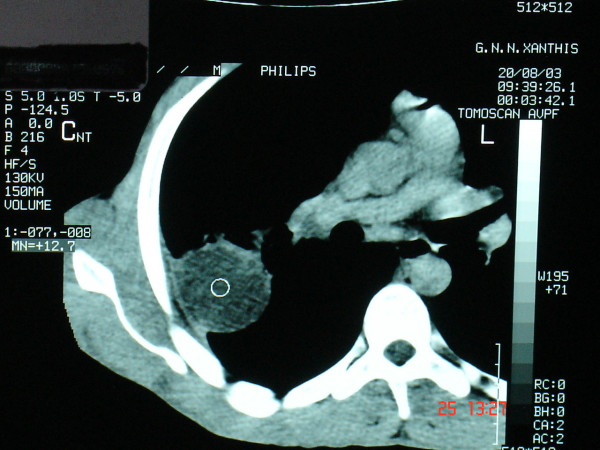
Computed tomography revealed a right lower lobe soft tissue density mass.

**Figure 3:**
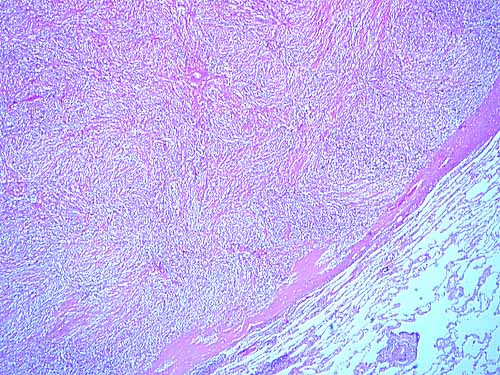
The mass was composed of fibroblasts, collagen and inflammatory cells mainly of lymphocytes and plasma cells (Hematoxylin-Eosin × 100).

His chest radiograph revealed a perbipheral right lung opacity, and computed tomography (CT) demonstrated a right lower lobe soft tissue density mass [Figures 1, 2]. Enlarged mediastinal lymph nodes were not identified. Bronchoscopy was negative. Transthoracic fine-needle aspiration under CT guidance was not diagnostic. A complete study of bone, brain and abdomen was also performed to assess the stage of a presumed bronchogenic carcinoma and no extrapulmonary involvement was noted. Surgery was carried out to obtain diagnosis and achieve cure. The patient underwent a right lower lobectomy and complete lymph node dissection through a right posterolateral thoracotomy. Macroscopically, the mass appeared well-circumscribed and yellowish in colour on cut section. Microscopically the mass was composed of fibroblasts, collagen and inflammatory cells mainly of lymphocytes and plasma cells (Figure 3). There was no mitotic activity. No microorganisms were detected even on special stains. The overall features suggested an inflammatory pseudotumor. All lymph nodes were negative for malignancy. The postoperative period was uneventful and the patient discharged home on postoperative day 10. The patient remained well and asymptomatic two years later.

## Discussion

Inflammatory myofibroblastic tumor of the lung is rare and its incidence is reported to be 0.04–1% of all pulmonary tumours [1]. Although these lesions can grow at a wide variety of other sites, they usually arise within the lung [3]. Pulmonary IMT is the most common lung tumor in patients younger than 16 years, and there does not appear to be a predilection for sex [4,5].

Common symptoms include cough, dyspnoea, fever, pleuritic pain and haemoptysis. A significant proportion of cases (30–70%) remain asymptomatic [4]. A history of upper respiratory tract infections or pneumonia is reported in approximately 30% of cases [6]. In our case, anorexia and hemoptysis were the main symptoms and a history of previous respiratory infection was also reported. Occasional cases of inflammatory pseudotumors of the lung are reported in which bacteria and fungi are isolated [7].

Previous studies have reported anaemia, elevated erythrocyte sedimentation rate, thrombocytosis and polyclonal hypergammaglobulinemia [8,9]. The first two findings were detected in our patient.

The radiologic features of inflammatory pseudotumors of the lung have been analyzed by Agrons et al [10]. Computed tomographic scan shows a nodule or a mass in approximately 90% of patients and multiple nodules in 5%. Secondary infiltration of hilum, mediastinum and airways occurs rarely. Calcification or cavitation is also reported but it is very infrequent [11]. Generally, computed tomography is not able to identify any specific features of inflammatory pseudotumors and all patients are eligible for surgery with suspected lung cancer as the diagnosis.

Needle biopsy has been suggested as a feasible approach to the diagnosis [12]. Fine needle aspiration shows a mixture of inflammatory cells, including plasma cells, fibroblasts and pneumocytes. Such findings are non-specific, given that inflammatory lesions of different origin can present the same picture. Moreover, inflammation and fibrosis can sometimes represent a reaction around a malignant tumor. Cerfolio et al proposed that these preoperative procedures are unnecessary and recommended complete resection for both diagnosis and treatment [1]. Attempted fine-needle aspiration cytology in our case also failed to establish diagnosis.

Gross pathology demonstrates that pulmonary inflammatory pseudotumors typically form a well-defined, firm, lobulated parenchymal nodule or mass with a whorled and often heterogeneous appearance on cut section. Histologically, IMT is composed of a variable inflammatory and mesenchymal cellular mixture including plasma cells, histiocytes, lymphocytes and spindle cells. Therefore, depending on the predominant cellular components, many synonyms for this disease have been described. Pettinato et al referred to this entity as IMT because the bulk of the lesion invariably consisted of not specific inflammatory cells but proliferative myofibroblasts and fibroblasts [13]. Most of the spindle cells are myofibroblasts, which show immunohistochemical staining for vimentin and smooth muscle actin and consistent ultrastructural features. The spindle cells commonly have low cellular atypia and no mitotic activity. The differential diagnosis of IMT is multifarious because of its variable cellular admixture. It includes malignant lymphoma, lymphoid hyperplasia, pseudolymphoma, plasmacytoma, malignant fibrous histiocytoma, sarcomatoid carcinoma, sclerosing haemangioma, sarcoma and chronic nodular pneumonitis [14]. These lesions can be differentiated by careful attention to cellular atypia, necrosis, mitotic activity, immunoreactivity or clonality [1,2].

The treatment of choice of inflammatory pseudotumor of the lung is surgery [15]. Wedge resection, if radical is suitable for curative purposes. When it is not technically feasible, the lesion is removed with major resection (lobectomy or pneumonectomy). In some cases, neighbouring anatomic structures (chest wall, diaphragm)also need to be excised. The effectiveness of radiotherapy, chemotherapy or steroids is uncertain [1,16]. Spontaneous regression of IMT has been reported only infrequently. In our case, a right lower lobectomy was performed with radical lymph node dissection and two years later the patient is in an excellent condition undergoing a stringent and prolonged follow up.

The prognosis of patients who undergo radical resection is excellent [1,7]. Nevertheless, relapse can occur even many years after resection and disease-related deaths are reported [17]. In recent years, inflammatory pseudotumor of the lung with considerable biologic aggressiveness and unfavourable evolution have been described with increasing frequency. Death is secondary to both local relapse with infiltration of the mediastinal organs and distant metastases [18].

In conclusion, IMT is a rare disease with similar characteristics to those of a true tumor. clinicians should retain an index of suspicion for the presence of this disease in young patients with symptoms or chest radiographs which suggest the presence of a malignancy. Surgical resection when possible, is recommended as the treatment of choice with an excellent outcome. Long-term follow up is imperative to detect recurrence.
